# Reevaluation of a classification system: stable and unstable odontoid fractures in geriatric patients—a radiological outcome measurement

**DOI:** 10.1007/s00068-022-01985-0

**Published:** 2022-05-21

**Authors:** Amelie Deluca, Florian Wichlas, Christian Deininger, Andreas Traweger, Ernst J. Mueller

**Affiliations:** 1 Institute of Tendon and Bone Regeneration, Spinal Cord Injury and Tissue Regeneration Center Salzburg, Paracelsus Medical University, Salzburg, Austria; 2 Department of Trauma Surgery, KABEG-Klinikum Klagenfurt a.W, Klagenfurt, Austria; 3 Department of Orthopedics and Traumatology, Salzburg University Hospital, Salzburg, Austria

**Keywords:** Odontoid fractures, Geriatric patients, Operative and non operative management

## Abstract

**Objectives:**

We carried out a retrospective cohort study to differentiate geriatric odontoid fractures into stable and unstable and correlated it with fracture fusion rates. Results are based on the literature and on our own experience. The authors propose that the simple Anderson and D’Alonzo classification may not be sufficient for geriatric patients.

**Methods:**

There were 89 patients ≥ 65 years who presented at our institution with type II and III odontoid fractures from 2003 until 2017 and were included in this study. Each patient was categorized with CT scans to evaluate the type of fracture, fracture gap (mm), fracture angulation (°), fracture displacement (mm) and direction (ventral, dorsal). Fractures were categorized as stable [SF] or unstable [UF] distinguished by the parameters of its angulation (< / > 11°) and displacement (< / > 5 mm) with a follow-up time of 6 months.

SFs were treated with a semi-rigid immobilization for 6 weeks, UFs surgically—preferably with a C1–C2 posterior fusion.

**Results:**

The classification into SFs and UFs was significant for its angulation (*P* = 0.0006) and displacement (*P* < 0.0001). SF group (*n* = 57): A primary stable union was observed in 35, a stable non-union in 10, and an unstable non-union in 8 patients of which 4 were treated with a C1/2 fixation. The overall consolidation rate was 79%. UF group (*n* = 32): A posterior C1–C2 fusion was carried out in 23 patients, a C0 onto C4 stabilization in 7 and an anterior odontoid screw fixation in 2. The union rate was 100%. Twenty-one type II SFs (91%) consolidated with a nonoperative management (*P* < 0.001). A primary non-union occurred more often in type II than in type III fractures (*P* = 0.0023). There was no significant difference in the 30-day overall case fatality (*P* = 0.3786).

**Conclusion:**

To separate dens fractures into SFs and UFs is feasible. For SFs, semi-rigid immobilization provides a high consolidation rate. Stable non-unions are acceptable, and the authors suggest a posterior transarticular C1–C2 fixation as the preferred surgical treatment for UFs.

**Level of evidence:**

Level III.

## Introduction

Odontoid fractures account for approximately 20% of the entity of cervical spine fractures in the adult population [3, 4]. However, they are the single most common observed cervical spine fracture in geriatric patients (≥ 65 years) [1, 2, 5, 6] and follow a bimodal population distribution with peaks in the early adulthood (high-energy trauma) and in the elderly (low-energy, minor trauma) [7, 8].

The so far established treatment protocols for odontoid fractures in the geriatric population remain controversial. A mortality rate of 26–47% has been reported for nonoperatively treated patients mainly due to respiratory-related complications from immobilization [9, 10].

In the literature, the reported rate of non-union for all treatment modalities varies greatly (2.4–82%) [10–16] and may be accounted for by various factors such as mechanism of injury, fracture type, direction of displacement, magnitude of angulation, blood-supply, osteoporosis, patients age, etc. [10, 12, 16–20]. Some authors consider a stable non-union as an acceptable outcome for the elderly [18, 50].

Surgical interventions, such as anterior odontoid screw fixation or posterior atlantoaxial arthrodesis are associated with significant complications in the elderly and are controversially discussed in the literature [21].

Harrop et al. and Yuan et al. state that anterior screw fixation in type II fractures is warranted in geriatric patients, as it offers the preservation of the C1–C2 rotatory motion and is less invasive [9, 22]. However, there are several reports on a high number of adverse events in the geriatric population and this operative procedure is more appropriate for younger patients [11, 12, 23, 24].

In contrast, posterior C1–C2 fixation reduces the rotatory motion but offers a high success rate with a bony union in the elderly with a low number of post-operative complications [21, 25–27].

In this observational, retrospective cohort study, we postulate that odontoid fractures in elderly patients can be differentiated into stable fractures (SFs) and unstable fractures (UFs) according to fracture angulation, displacement, and gap. The proposed fracture classification by Anderson and D’ Alonzo or its modifications are not suitable for type II fractures in the elderly due to high operative complication rates. Hence, a suitable alternative, as outlined in this study, represents the division of geriatric odontoid fracture into SFs and UFs, leading to safe outcomes. For this purpose, we analyzed non-operatively treated SFs and operatively treated UFs of the odontoid in the elderly and compared the two of them.

## Materials and methods

### Study design

This is a retrospective study of clinical and radiological outcomes in a cohort of geriatric patients (≥ 65 years) with acute type II and III odontoid fractures (< 21 days since accident) managed operatively and non-operatively.

Extracted data after management included: fracture type, -angulation, -displacement, -gap, number and severity of initial neurological deficits, age, initial treatment modality, fracture healing, mortality, associated injuries, and sex.

### Study population

Medical records and CT scans of the cervical spine of 211 consecutive patients with odontoid fractures were analyzed who presented at our Level I tertiary-care center between May 2003 and December 2017.

Seventy-four patients were excluded because they were under 65 years old, presented with missing/incomplete radiologic/clinical data, had suffered a polytrauma (ISS > 16) [28], showed anatomical/congenital abnormalities of the C1/2 region, suffered from metastatic/rheumatoid disease, or fractures were of unknown age.

**Fig. 1:**
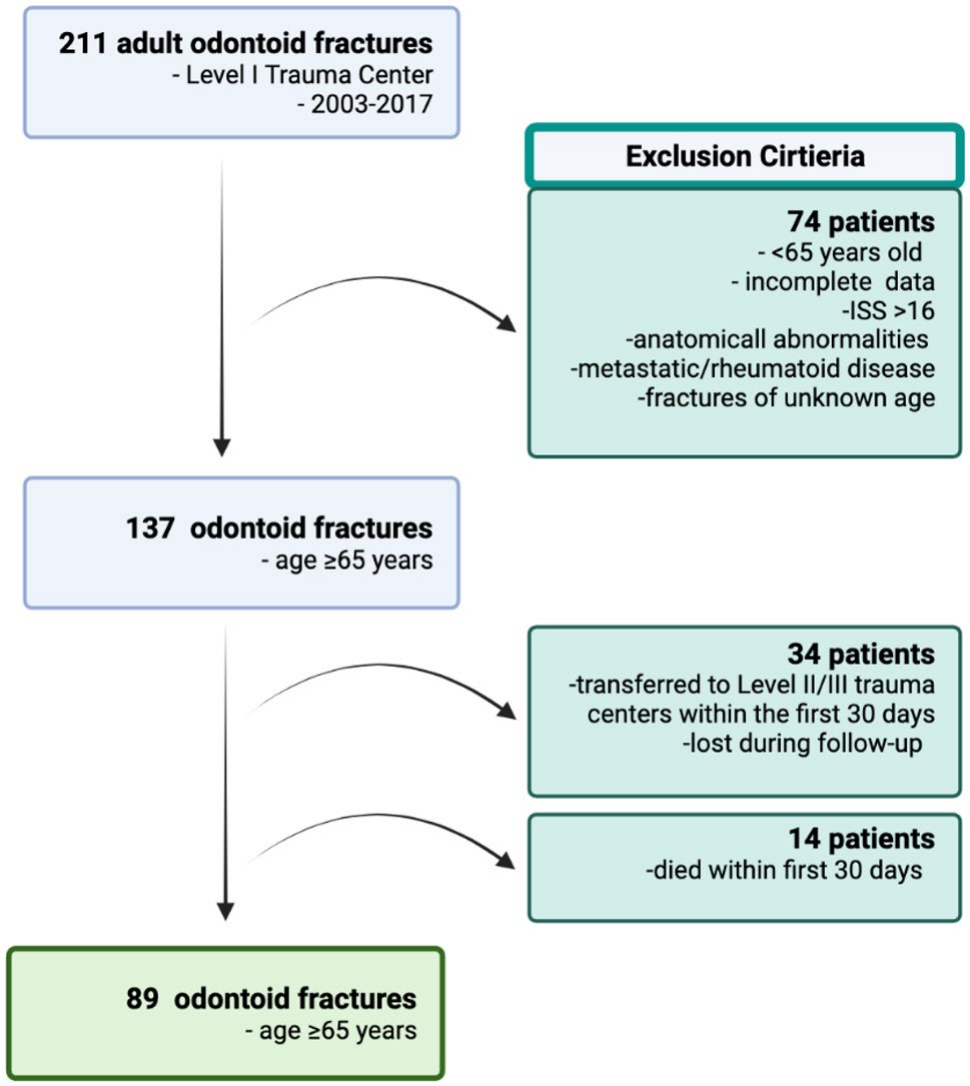
Study flow diagram: during the study period, inclusion criteria were met by 89 elderly patients with an odontoid fracture

The remaining 137 patients were included aged 65 years or older and sustained a type II (74) or III (63) odontoid fracture (International Classification of Diseases, Ninth Revision, code 805.02) (Fig. 1).

### Radiological fracture evaluation

Fracture evaluation was solely based on CT scans (Aquilion One; Toshiba, Canon Medical Systems, Vienna, Austria and Dual Source Somatom; Siemens, Munich, Germany) and classified according to Anderson and D’Alonzo [14]. Obtained measurements included fracture angulation (degrees), displacement (mm), and gap (mm) as well as a treatment modality.

**Fig. 2:**
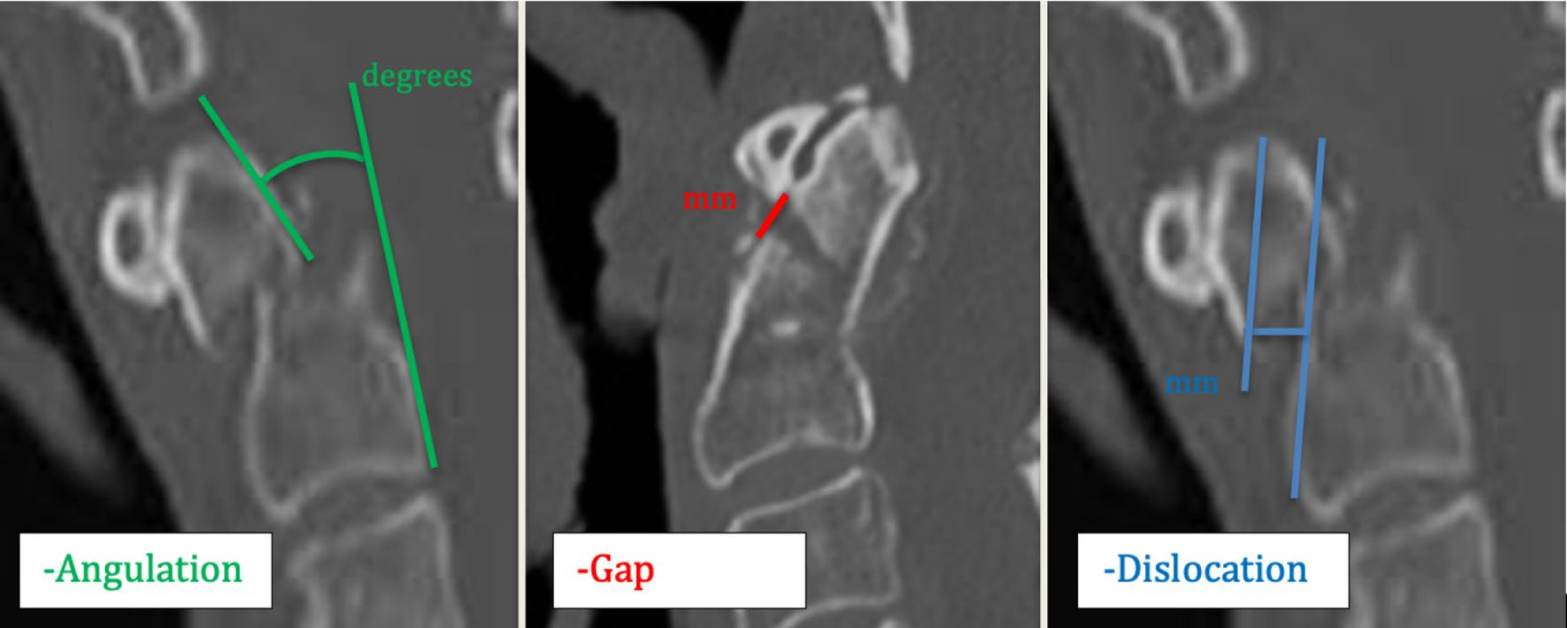
Odontoid fracture classification according to ventral or dorsal dislocation (blue), angulation (green) or gap (red)

Encountered odontoid fractures were differentiated into SFs and UFs, according to previously published clinical cutoff points [19, 20, 29, 30]. SFs displayed an initial ventral or dorsal displacement of < 5 mm and an angulation of < 11°. All other fractures were classified as UFs. The obtained measurements for the gap (mm) have been neglected as overall 63 fractures (71%; 45 SFs, 18 UFs) displayed a gap of 0.00 mm (Fig. 2).

Measurements were performed using the hospital’s web-based picture archiving and communication system, which is integrated into the medical record system, IMPAX EE (Agfa Health Care, Bonn, Germany). An additional MRI examination was carried out on 8 patients with neurological deficits either upon arrival or within 12 h after symptoms occurred.

### Treatment protocol based on stable and unstable radiologic fracture evaluation

SFs were treated with a semi-rigid immobilization, Vista collar (Vista Orthese Aspen Medical Products, Griesheim, Germany) or Philadelphia collar (Philadelphia Cervical Collar Company, Thorofare, New Jersey, USA) for a minimum of 6 weeks.

UFs were treated operatively. The same three surgeons, with consistent surgical approaches, performed all procedures: An altered posterior transarticular C1–C2 fixation [12, 31] according to Magerl and Seemann [43, 44].

If anatomic landmarks prohibited a posterior C1–C2 arthrodesis due to extensive cystic lesions a C0 onto C4 stabilization was done.

An anterior odontoid screw fixation was performed if prone positioning was not possible due to concomitant spinal injuries. (Appendix 1).

### Clinical and radiological follow-up

Follow-up monitoring was done in all patients (SFs and UFs) at the time of hospital discharge or suture removal, as well as at 6, 12- and 24 weeks post-treatment initiation.

Routine assessments consisted of clinical and radiographic evaluation (antero-posterior, lateral, open-mouth view, and if possible dynamic X-ray evaluation).

Successful fracture consolidation was defined as signs of osseous trabeculae crossing the fracture site in the absence of sclerotic margins along the fracture line with no change in neurology. Two individual and independent surgeons observed the X-rays at the given post-trauma intervals to state whether osseous trabeculae crossing at the fracture site was present. If conventional X-ray was not conclusive and fracture consolidation or stability could not be determined, or the patient complained about ongoing pain, an additional CT scan was carried out (62%) in the SF group. All patients in the UF group have been evaluated with a CT scan post-surgery to check upon fracture reduction and implant positioning to ensure adequate fracture healing.

### Statistical analysis

Collected data are expressed as mean ± standard deviation. Frequency distributions and summary statistics were calculated for demographics and fracture types. For categorical variables, cross-tabulations were generated, and Fisher exact tests were used to compare distributions. Difference in survival between the SFs and UFs were modeled using the Cox proportional hazard model including adjustments for age and sex. A 30 day Kaplan–Meier survival plot was created for mortality comparison between SFs and UFs. The level of statistical significance was set at *P* < 0.050. Statistical analysis was conducted using GraphPad Prism 9.0.0 (San Diego, CA, USA).

## Results

### Patients characteristics

This study included 137 patients aged 65 years or older (60 male, mean age 80 ± 8 years; range 66–94 years). Of these, 34 patients (24.8%; 26 SFs, 8 UFs) were transferred to Level II/III trauma centers within the first 30 days or lost during follow-up and hence, excluded. Patients were transferred to a different hospital after initiating treatment due to closer proximity to their hometown. Within 30 days of initial treatment, 14 patients died (10.2%, 7 SFs; 7 UFs).

In the remaining 89 patients, there was no significant difference in the proportion of male *versus* female (*P* = 0.499) (Table 1).

**Table 1:** Descriptive data analysis of stable and unstable dens fractures in geriatric patients

	Type II	Type III	Angulation (Degree)	Displacement (mm)	Gap (mm)
Stable Dens Fractures [SF] *n* = 57	23	34	8.17 ± 12.17	1.81 ± 2.33	0.49 ± 1.10
Unstable Dens Fractures [UF] *n* = 32	20	12	19.47 ± 14.83	5.36 ± 3.68	1.14 ± 1.56
Anterior Screw Fixation *n *=2	1	1	15.50 ± 2.12	4.50 ± 3.12	1.59 ± 1.15
Posterior C1–C2 Fixation *n* = 23	15	8	19.78 ± 15.18	4.89 ± 3.84	1.09 ± 1.58
C0 onto C4 Stabilization *n* = 7	4	3	19.57 ± 17.53	7.43 ± 2.99	0.80 ± 1.44

**Fig. 3:**
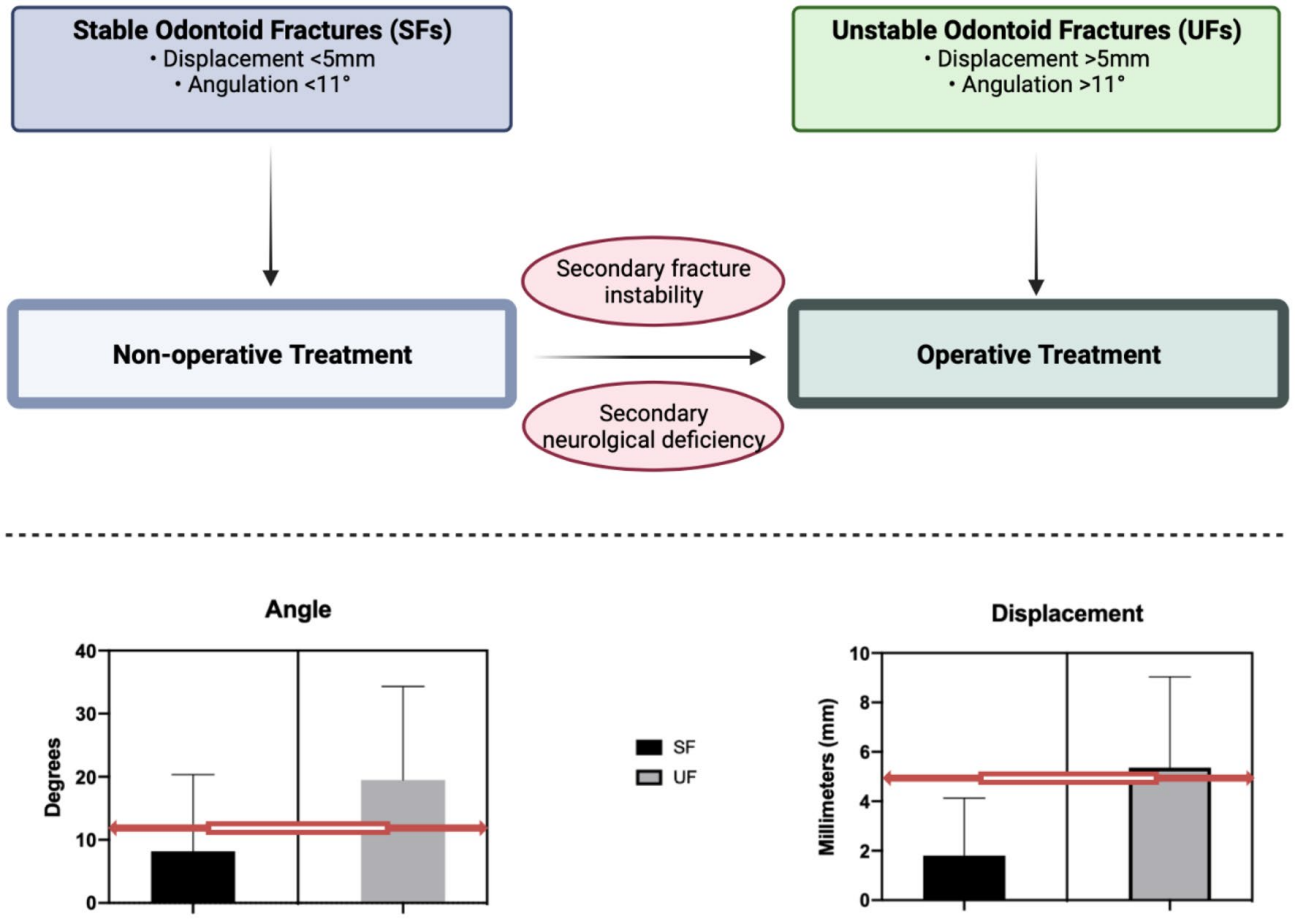
Treatment algorithm of odontoid fracturs in the elderly (top). Classification into SFs and UFs based on target cut off points (red line)—angulation and displacement (bottom)

A significant difference between the 2 groups, SFs and UFs according to fracture angulation (*P* = 0.001) and displacement (*P* < 0.0001) could be observed as depicted in Fig. 3. The average follow-up time of the remaining 89 patients, 57 SFs and 32 UFs, was 6.9 ± 12.3 and 6.9 ± 10.7 months, respectively.

### Radiographic outcome: SFs

**Fig. 4:**
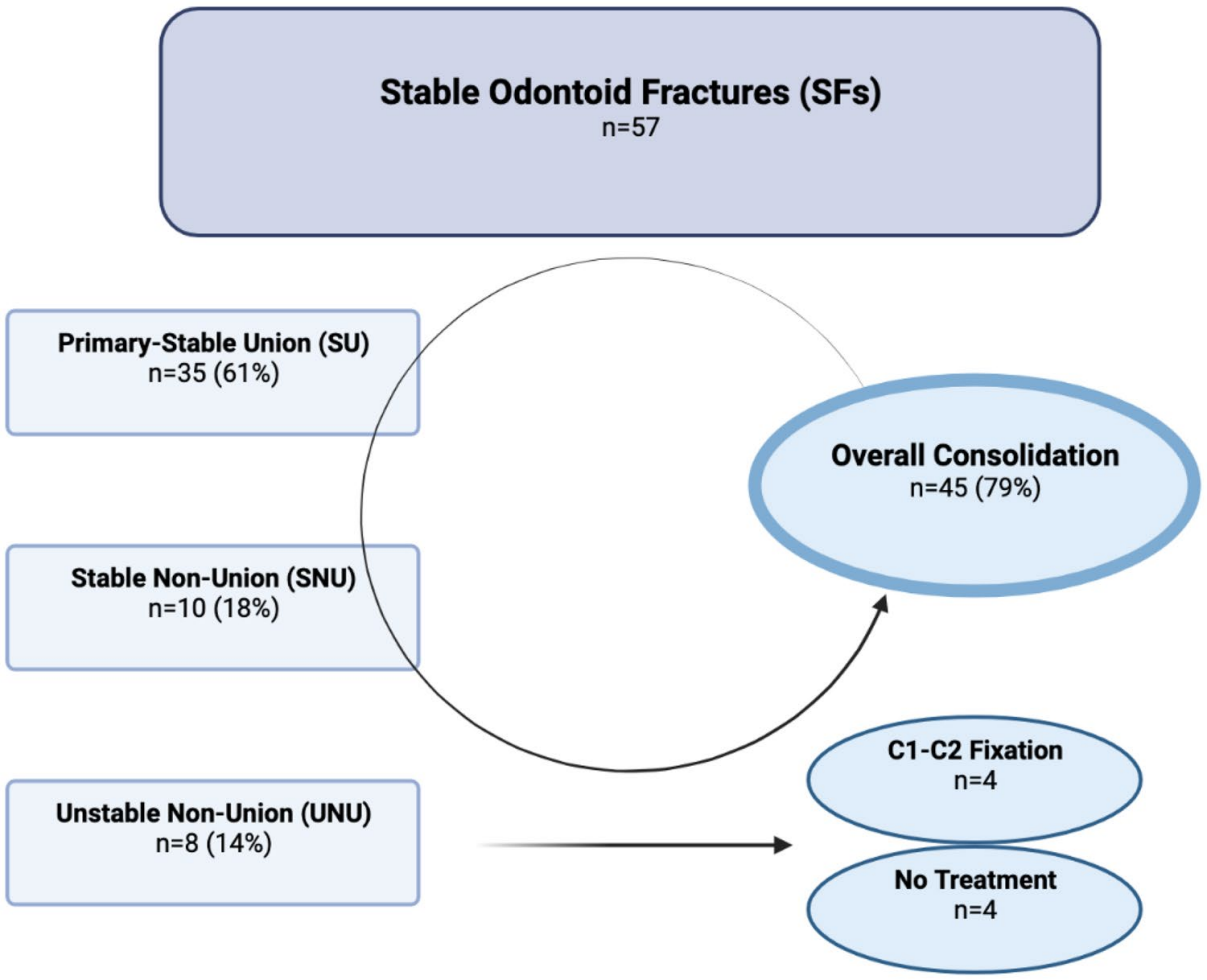
Summarized data and treatment protocol of stable odontoid fractures in the elderly

As depicted in Fig. 4, 57 SFs (23 type II, 34 type III) were identified after 6 months and divided into subgroups: stable union (SU), stable non-union (SNU) and unstable non-union (UNU). Union was defined as bony consolidation of the fracture and stability was determined by the absence of a secondary displacement (< 2 mm). UNU was characterized by the absence of osseous bony union and a secondary odontoid displacement (> 2 mm).

A SU was observed in 39 patients (12 type II, 27 type III). A primary SU occurred in 35 patients (11 type II, 24 type III) and a secondary-SU with consolidation after secondary dislocation in 4 patients (2 type II, 2 type III) after 6 months.

A Stable Non-Union (SNU) was observed in 10 patients (7 type II, 3 type III).

An Unstable Non-Union (UNU) occurred in 8 patients of which 4 could not undergo surgery due to comorbidities or they did not wish to be operated. The other 4 fractures were secondarily stabilized with a C1–C2 posterior fixation (2 type II, 2 type III) proceeding to bony fusion.

Overall, 79% of encountered SFs fully consolidated (*P* < 0.0001).

### Radiographic outcome: UFs

Radiographic evaluation showed bony union at the site of posterior C1–C2 Arthrodesis in all patients that initially presented with UFs (15 type II, 8 type III fractures) [12, 31]. This included one patient (4%) with screw malpositioning that had to be surgically revised and the osseous union was attained within three months. A single screw breakage was observed in 3 patients, but all achieved full consolidation with no neurological deficits, and none needed revision.

Due to extensive cystic lesions in the odontoid, a C0 onto C4 Stabilization was used to stabilize the fracture (4 type II, 3 type III) and all consolidated.

Due to a concomitant luxated C6-C7 fracture with simultaneous ventral plate stabilization in one patient and a traumatic brain injury in the other, an Anterior Odontoid Screw Fixation (1 type II, 1 type III) was the surgical method of choice.

**Fig. 5:**
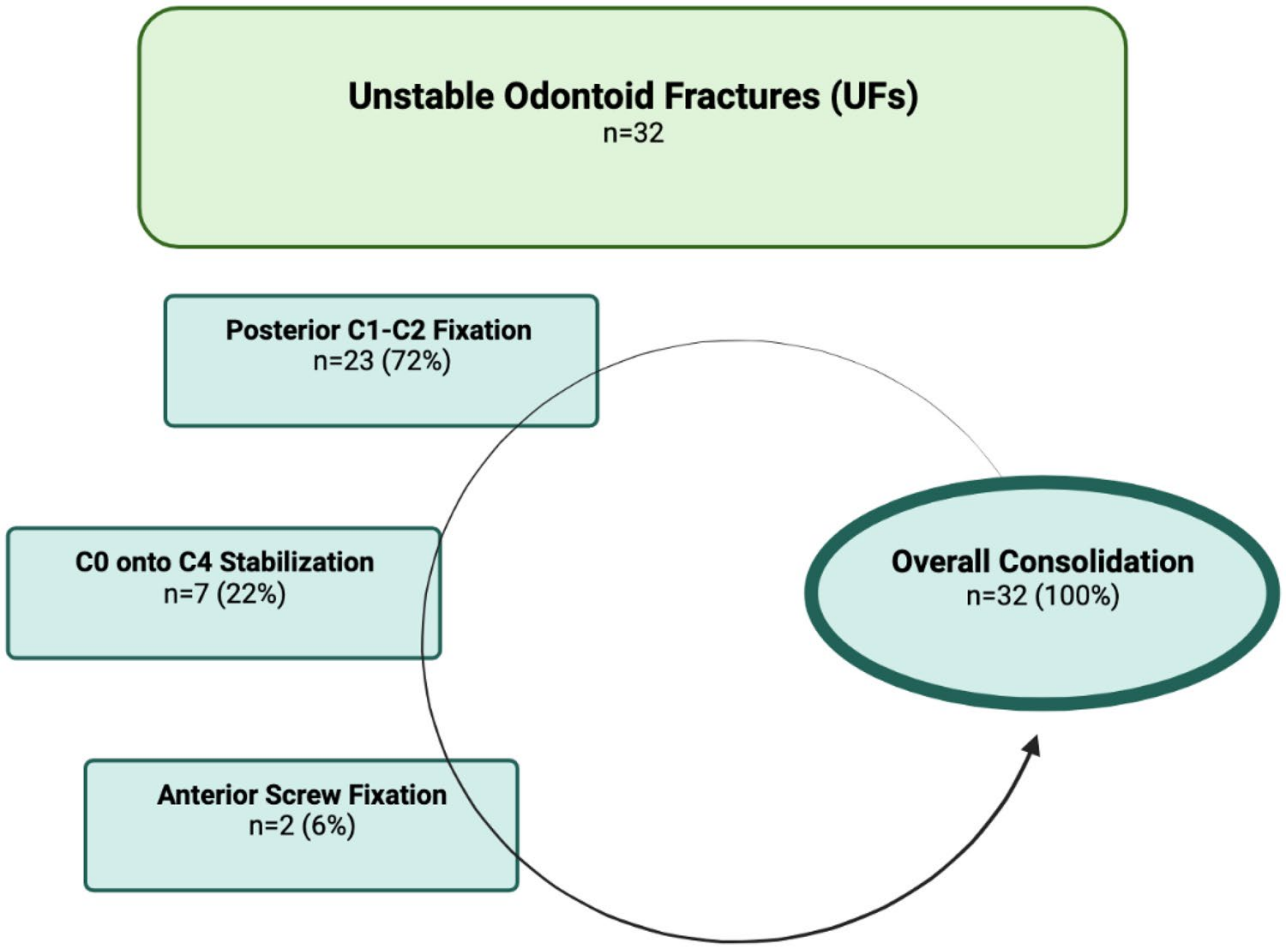
Summarized data and treatment protocol of unstable and operatively treated odontoid fractures in the elderly

The overall consolidation rate of all UFs was 100% (Fig. 5).

### Type II fractures: SFs versus UFs

**Fig. 6:**
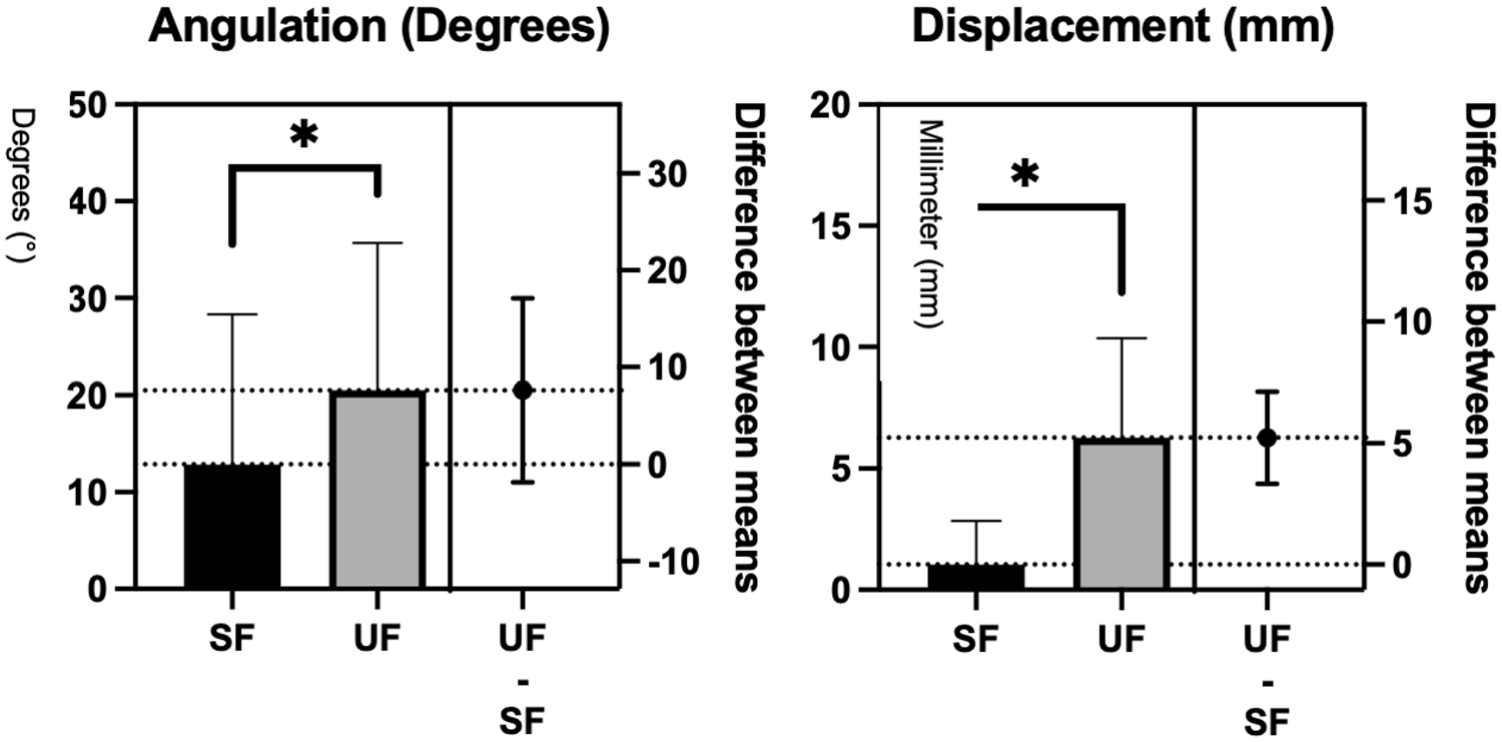
Comparison of type II odontoid fractures in the SF und UF group according to angulation and displacement. *P < 0.01

A total of 43 type II fractures (23 SF, 20 UF) were analyzed in this cohort. In the SF group, 23 type II fractures were treated nonoperatively and resulted in either a primary non-union (SNU *n* = 7, UNU *n* = 4) or a SU (*n* = 12). In the UF group, 20 type II fractures were treated operatively leading to a stable union (100%). Ninteen out of the twenty-three type II fractures (83%) in the SF group healed as a SU or SNU with non-operative management compared to type II fracturs in the UF group. This is significant (*P* < 0.001) and indicates that not all type II fractures need to be operated on and to be considered as stable or to reach bony union. Figure 6 depicts that there is a significant difference (*P* = 0.0115) in angulation (mean SF: 12.87° ± 15.45; mean UF: 20.50° ± 15.19) compared to its theoretical mean cutoff point of 11 degrees between type II SFs and UFs. The significance of type II fractures between both groups also applies to its dislocation (mean SF: 1.04 mm ± 1.80; mean UF: 6.28 mm ± 4.08; *P* < 0.0001) compared to its theoretical mean cutoff point of 5 mm.

### Case fatality rate over 30 days

Patients who died within 30 days after diagnosis were not included in the summarized statistic above and evaluated separately. Overall, 14 patients died (17 SFs, 7 UFs).

In the SF group (5 type II, 2 type III), 6 died shortly after the initial injury due to cardiac arrest and/or respiratory insufficiency. One patient deceased within 3 weeks due to multi-organ failure.

Of the 7 patients in the UF group (4 type II, 3 type III), 6 died due to cardiorespiratory insufficiency and one patient due to multiorgan failure. All deceased patients were treated with a posterior transarticular C1–C2 fixation. One of these patients presented initially with tetraplegia.

The overall case fatality rate (CFR) does not reflect a significant difference between SFs and UFs (*P* = 0.3786), between males (*n* = 7) and females (*n* = 7) (*P* > 0.9999) or type II (*n* = 9) and III fractures (*n* = 5) (*P* = 0.3896). The hazard ratio of death within the first 30 days of presentation in the SF group compared to patients in the UF group was 1.47 (95% CI = 0.44–4.87).

### The Effect of neurological deficits on the CFR

Initial neurological deficits were observed in 14 patients (Table 2) and 5 deceased within 30 days. All deceased patients were quadriplegic (2 SFs with nonoperative treatment due to cardiac resuscitation and respiratory insufficiency, 3 UFs with C1/2 fixation).

**Table 2:** Initial neurological deficits, treatment and outcome according to the Frankl classification [51]

Age	Anderson	FRANKL	Initial Neurology	Treatment	Outcome
87	3	E	Dysesthesia left UE	Conservative (SF)	No deficits
82	2	A	Quadriplegia sub C2	Conservative	Deceased
65	3	A	Quadriplegia sub C2	Conservative	Deceased
77	3	A	Incomplete Paraplegia sub C2	C1–C2 Fixation (UF)	Incomplete Paraplegia sub C2
85	3	C	Bilateral Paresis UE	C1–C2 Fixation (UF)	Bilateral Paresis
90	2	B	Hemiparesis right UE	C1–C2 Fixation (UF)	Dysesthesia left UE
75	2	E	Dysesthesia UE	C1–C2 Fixation (UF)	Dysesthesia UE
88	2	A	Quadriplegia sub C2	C1–C2 Fixation (UF)	Deceased
79	2	A	Incomplete Paraplegia sub C2	C1–C2 Fixation (UF)	Incomplete Paraplegia sub C2
81	2	E	Dysesthesia UE	C0 onto C4 Stabilization (UF)	No deficits
79	2	B	Tetra paresis sub C2	C1–C2 Fixation (UF)	Deceased
81	2	D	Motor deficits C6, C7	C0 onto C4 Stabilization (UF)	No deficits
72	2	E	Dysesthesia UE	C1–C2 Fixation (UF)	No deficits
74	3	A	Quadriplegia sub C2	C1–C2 Fixation (UF)	Deceased

*UE *upper extremities

A dorsal fracture dislocation was observed in 9 patients (*P* = 0.5055). Four out of the five patients who died suffered from a dorsal dislocation as well (*P* = 0.4000). An MRI examination was carried out in patients whose vital functions permitted the investigation.

## Discussion

The available literature reports on different treatment modalities for type II and III odontoid fractures in the elderly but lacks specific guidelines for its application.

Observed radiographic parameters (angulation, displacement) are important to decide whether an operative treatment is warranted and are predictive of treatment outcomes [28, 45]. Karamian et al. demonstrated that the obtained measurements via CT scans, as used in our study, are reliable [46]. The classification of odontoid fractures into SFs and UFs based on CT scan measurements was constituted on previously mentioned cut-off points [19, 20, 29, 30] and were significant in the measurements obtained in our study. According to our calculation and further stratification, to classify odontoid fractures not based on the previously published guidelines of Anderson and D’ Alonzo, but rather into SFs and UFs is justifiable.

Semi-rigid immobilization with a cervical collar is biomechanically superior to the halo orthoses with a significantly lower device-associated complications [18, 19, 29, 31] and was used as the treatment of choice. Overall, 35 patients (61%) in the SF group advanced to a SU which is within the range reported by others [48]. A SNU was seen in 10 patients (18%), with no observed neurological deficits and no instability at the fracture site (dynamic X-ray evaluation) [18, 32–34]. According to our results, we agree that stable fibrous union is an acceptable outcome in geriatric odontoid fractures [49]. The evaluation of fracture union is one of the most important and fundamental clinical determinations made in orthopedics. If the observed X-rays showed signs of fracture consolidation, an additional CT scan evaluation was omitted in the SF group. If there were any signs of non-union or pain, a CT scan was carried out to evaluate fracture fusion rates. In summary, 79% presented with a stable fracture and we concur with many authors that fracture stability is the main goal to pursue with or without proper osseous union [12, 18, 32, 49].

Posterior transarticular C1–C2 fixation resulted in 100% bony union (*n* = 23). It was our primary choice of treatment in the UF group [34], and the observed rate of consolidation is concurrent with published literature results [27, 35]. The difficulty in placing screws has been reported previously in up to 26% of patients [36]^.^ In our case study, obstacles were observed due to an excessive thoracic kyphosis or a high-riding vertebral artery, as well as destructive cystic lesions [29, 38]. Several authors reported decreased functional results with a considerable impairment in cervical spine motion as well as chronic pain symptoms [36–38].

In contrast to a posterior arthrodesis, direct anterior screw fixation of the dens preserves the C1–C2 rotation [23]. Based on our own experience and on published reports, it is the preferred treatment in patients younger than 65 years of age [11] with a type II fracture, no osteoporosis, and with a horizontal or an anterior–posterior declining fracture line [12, 24, 30, 34].

Smith et al. reviewed trends in the surgical management of type II fractures over 20 years in their institution and noted posterior methods for fracture stabilization were used much more than anterior odontoid screw fixation [39]. The latter was associated with significantly higher rates of postoperative pneumonia, swallowing dysfunction, and increased technical problems [7, 24, 30, 39, 40]. Osti et al. analyzed failure after anterior screw fixation in geriatric patients and found a significant association between both failure-to-heal and age, as well as failure-to-heal and severity of degenerative changes, such as cystic lesions in the odontoid [41].

According to our radiological UF results, posterior atlantoaxial screw fixation is a good alternative in geriatric patients due to a significantly higher rate of bony union. We further propose that an anterior screw fixation should only be carried out in geriatric patients when a C1–C2 fusion is not suitable such as in patients with traumatic brain injury when supine positioning is not achievable or concomitant surgical approaches have to be carried out.

On the one hand, Anderson and D’Alonzo referred to surgical fracture stabilization of all type II fractures. Based on our observed results by delineating fractures into stable and unstable, not all type II fractures have to undergo surgery to achieve consolidation. Out of 23 nonoperatively treated type II fractures, 21 attained a primary healing or a stable pseudoarthrosis with no neurological dysfunction (*P* < 0.001). Only one patient (4%) developed delayed-onset myelopathy 5 days after injury and had to be managed operatively which is not a well-established risk factor in the literature [14, 18, 34, 35].

On the other, our results are concurrent with Anderson and D’Alonzo that non-operatively treated type II fractures produce a primary non-union more often than type III odontoid fractures (9 type II, 4 type III) [14]. Hence in the SF group, a SU or SNU in geriatric type II odontoid fractures are an acceptable outcome.

The 30-day case fatality rate showed a hazard ratio of 1.47, indicating a slightly higher rate of survival among patients that were treated nonoperatively (95% [confidence interval] CI = 0.44–4.87, *P* = 0.4064), but was not significant as described by other authors [47]. The highest rate of fatality in patients with neurological deficiencies could be observed in geriatric patients with quadriplegia below C2. There was no significant difference in survival in patients with neurological deficits that were operated (HR = 0.08; CI = 0.00–1.82; *P* = 11.38).

In our study, neurological deficits were documented in 14 patients of whom 9 patients presented with a dorsal fracture dislocation (64%). These results compare to the findings of Ryan and Taylor [42] and Mueller et al. [12] indicating that a higher incidence of concomitant spinal cord injury is found in patients that are older than seventy years with a dorsal fracture dislocation. Even though there seems to be enough space in the spinal cord canal, the cord is at greater risk with dorsal fracture dislocation [12].

This retrospective, non-randomized study is not without limitations. Only analyzed data collected from documented electronic records and a median follow-up time of 6 months. It was not possible to obtain a functional dislocation measurement in mm for the entire cohort (lateral flexion–extension radiographs), and therefore the data could not be included. There was no control group, the degree of osteoporosis was not determined, and this study was conducted at a single designated trauma center without randomization.

In the geriatric population, patient’s health considerations, such as osteoporosis/altered bone mineral density and cardiopulmonary comorbidities play an additional role in deciding upon the accurate treatment modality [12] and should be incorporated before an operative procedure is carried out.

## Conclusion

To differentiate odontoid fractures into SFs and UFs is feasible and showed valuable results. The authors suggest that type II fractures can be treated nonoperatively with a semi-rigid immobilization if they are classified as SFs. If a fracture classifies as an UF, operative treatment is warranted, and we suggest using a posterior C1–C2 fixation in geriatric patients as it showed a 100% fusion rate. There is no necessity for all type II fractures to be operatively stabilized if the fractures are delineated correctly into SFs and UFs. Overall, a SNU is acceptable in the geriatric population.

## Appendix 1: Concomitant spinal injuries

**Table:**

Age	Concomitant injuries of the cervical spine
83	Atlas arch fracture
72	C2 pedicle fracture
78	C2 pedicle fracture
85	Atlas arch fracture
87	Osteolysis
90	Atlas arch fracture
72	Atlas arch fracture
91	Jefferson fracture
65	Atlas arch fracture
67	Articular process abruption C2
80	Atlas arch fracture
76	C2-abruption proc. Spinosus
82	Atlas arch fracture
81	C2-abruption proc. Spinosus
82	C4-right arch fracture
71	Atlas arch fracture
72	Atlas arch fracture
92	Atlas arch fracture
88	Atlas arch fracture
91	Atlas arch fracture
79	C2-abruption proc. Spinosus
84	Atlas arch fracture
88	Atlas arch fracture
92	C2 pedicle fracture
70	Cystic lesion
74	Cystic lesion
72	C2 pedicle fracture
